# Supplementary material for: Engineering Inorganic Pyrophosphate Metabolism as a Strategy to Generate a Fluoride-Resistant Saccharomyces cerevisiae Strain
Source: Microorganisms. 2025 Jan 21;13(2):226. doi: 10.3390/microorganisms13020226 (PMC11857102; doi:10.3390/microorganisms13020226)
Supplement: Supplementary file 1 [file microorganisms-13-00226-s001.zip › Legends to supplementary figures.docx]

**Figure S1. Phylogenetic relationships of family I sPPases from fungi and animals.** A molecular phylogenetic analysis of family I sPPase paralogs (nucleo-cytosolic and mitochondrial isoforms) of fungi, protists and metazoans (animals) was performed. A distance (Neighbor-Joining) phylogenetic tree obtained from an alignment of amino acid sequences generated by CLUSTAL X and Sea View v5.2 programs is presented. PT, presence of N-terminal transit peptide, indicating organelle localization; *M*, mitochondrial enzymes. Sequences are identified by their UniProtKB accession numbers or their genome/transcriptome project code numbers. The family I sPPases used in this work are shown boxed in bold. Numbers in selected nodes are bootstrap values based on 100 replicates. Scale bar indicates number of changes per amino acid site.

**Figure S2. Molecular phylogeny of DHH-DHHA2 phosphoesterases (family II sPPases, PPX1-like exopolyphospha-tases and PRUNE-lke proteins).** These proteins are found in bacteria, archaea, protists and metazoa. Amino acid sequences were aligned using CLUSTALX to generate a phylogenetic distance tree (NJ) with the MEGA4 program. The bacterial family II sPPase used in this work (from *Strep. mutans*) is shown boxed in bold. Numbers in selected nodes are bootstrap values based on 1,000 replicates. Scale bar indicates number of changes per amino acid site. Family II sPPases occur in some Gram+ bacteria and Firmi-cutes, archaea and in some marine protists which may have acquired them by horizontal gene transfer (HGT) from prokaryotes.

**Figure S3. Phylogenetic relationships of ion-translocating mPPases.** A molecular phylogenetic analysis of the three main classes of mPPases, namely K+-dependent H+- and Na+-PPases and K+- independent H+-PPases of prokaryotes, protists and the green lineage (from microalgae to higher plants) was performed. A distance (Neighbor-Joining) phylogenetic tree obtained from an alignment of amino acid sequences generated by CLUSTAL X and Sea View v5.2 program is presented. The three classes of mPPases conform well-defined clusters, which are clearly divergent. Sequences are identified by their UniProtKB accession numbers or their genome/transcriptome sequencing projects code numbers. The mPPase MVP used in this work is shown boxed in bold. Numbers in selected nodes are bootstrap values based on 1,000 replicates. Scale bar indicates number of changes per amino acid site.
